# Corrigendum to “Social Recommendation System Based on Hypergraph Attention Network”

**DOI:** 10.1155/2022/9841237

**Published:** 2022-07-06

**Authors:** Zhongxiu Xia, Weiyu Zhang, Ziqiang Weng

**Affiliations:** School of Computer Science and Technology, Qilu University of Technology (Shandong Academy of Sciences), Jinan, Shandong 250353, China

In the article titled “Social Recommendation System Based on Hypergraph Attention Network” [1], the authors would like to clarify that the article reports similar methods and techniques as used by Yu et al. [2]. The second paragraph in Section 2.3, “Hypergraph in Recommender Systems,” should be replaced as follows with the addition of the missing reference 49 [2].

## 1. Original

Although hypergraph neural network shows the potential of high-order relational modeling in many fields, there is little work in the field of recommendation system, and only a few research works combine these two topics. HyperRec [38] uses the advantages of HGCN to recommend the next item for users. DHCN [39] models the session data as a hypergraph, and then extracts the feature information of the session by using the dual-channel hypergraph convolution network, so as to infer the next item in the session. SHARE [40] is different from DHCN in that it uses the hypergraph attention network (HGAT) [32], which can flexibly aggregate the context information of related items in the session to generate item embedding. However, these recommendation methods do not make use of social relations, resulting in unsatisfactory recommendation results. Therefore, we integrate social networks into the recommendation system, and exploit the potential information of users by using the characteristics of hypergraph that can capture complex high-order relations.

## 2. Revised

Although hypergraph neural network shows the potential of high-order relational modeling in many fields, there is little work in the field of recommendation system and only a few research works combine these two topics. HyperRec [38] uses the advantages of HGCN to recommend the next item for users. DHCN [39] models the session data as a hypergraph and then extracts the feature information of the session by using the dual-channel hypergraph convolution network, so as to infer the next item in the session. SHARE [40] is different from DHCN in that it uses the hypergraph attention network (HGAT) [32], which can flexibly aggregate the context information of related items in the session to generate item embedding. MHCN [49] uses hypergraph convolutional network technology to mine users' potential information by leveraging high-order user relations. However, these recommendation methods do not exploit the hypergraph attention network to capture users' social relations, resulting in unsatisfactory recommendation results. Therefore, we integrate social networks into the recommendation system and exploit the potential information of users by using the characteristics of hypergraph attention that can flexibly capture complex high-order relations.
